# Eye-hand coordination during robotic prostate cancer surgery – how surgical vision reflects on instrument kinematics and vice versa

**DOI:** 10.1007/s11701-026-03499-y

**Published:** 2026-05-28

**Authors:** Kateryna Pirkovets, Vera A. Ottens, Matthias N. van Oosterom, Henk G. van der Poel, Fijs W.B. van Leeuwen

**Affiliations:** 1 Interventional Molecular Imaging Laboratory, Leiden University Medical Center, Leiden, The Netherlands; 2 Department of Urology, Netherlands Cancer Institute – Antoni van Leeuwenhoek Hospital, Amsterdam, The Netherlands; 3 Department of Urology, Amsterdam University Medical Center, VUmc, Amsterdam, The Netherlands

**Keywords:** Robotic-surgery, Surgical performance evaluation, Endoscopic vision, Kinematic assessment, Fluorescence guided surgery, Lymph node dissection

## Abstract

During robot-assisted radical prostatectomy (RARP), vision drives the (tele)surgical actions. As such, endoscope movements can be considered a performance-defining parameter during robotic surgery. While instrument kinematics have been the subject of research, it remains unclear how steps in the surgical procedure are reflected in endoscope kinematics, and vice versa. Using the AP4000 mechanical instrument movement recorder, we digitized the x-, y-, and z-trajectories of the endoscope and the 3 robotic arms holding the dissecting instruments and synchronized these data with the endoscopic video feed. To obtain a real-life representation, these evaluations were performed in a clinical pilot setting with one expert surgeon; 4 RARP procedures of which 2 were accompanied by an ICG-fluorescence-guided pelvic lymph node dissection. Perhaps the most obvious endoscope movement observed was related to cleaning. Overall, we found that the step-averaged endoscope kinematic vectors, computed across all standardized features, differed between surgical steps. Hereby endoscope movements coordinated tightly with the activity of the dissecting instruments. The use of fluorescence impacted both endoscope and instrument kinematics. These new insights suggest that endoscopic camera control reflects the dynamics of the surgeon’s perceptual demands. In terms of clinical impact, an enhanced understanding of (expert) endoscope use could not only help surgeons better interpret their own actions, but also help optimize training programs aimed at improving surgical proficiency.

**Supplementary Information:**

The online version contains supplementary material available at 10.1007/s11701-026-03499-y.

## Introduction

Spencer noted in 1978, “A skillfully performed operation is about 75% decision-making and 25% dexterity.” [1] With regard to instrument dexterity, technological developments in robotic-assisted laparoscopic procedures - particularly the introduction of wristed, steerable instruments – have focused on enhancing freedom of movement in procedures such as intra-abdominal prostate cancer resections [2]. On the other side, during surgery the perception of a highly interactive environment forms the foundation for on-the-fly decision-making [3]. As such, perception-enhancing technologies are needed to advance the surgeon’s ability to make informed decisions with regards to the technical execution of the resection.

**Fig. 1:**
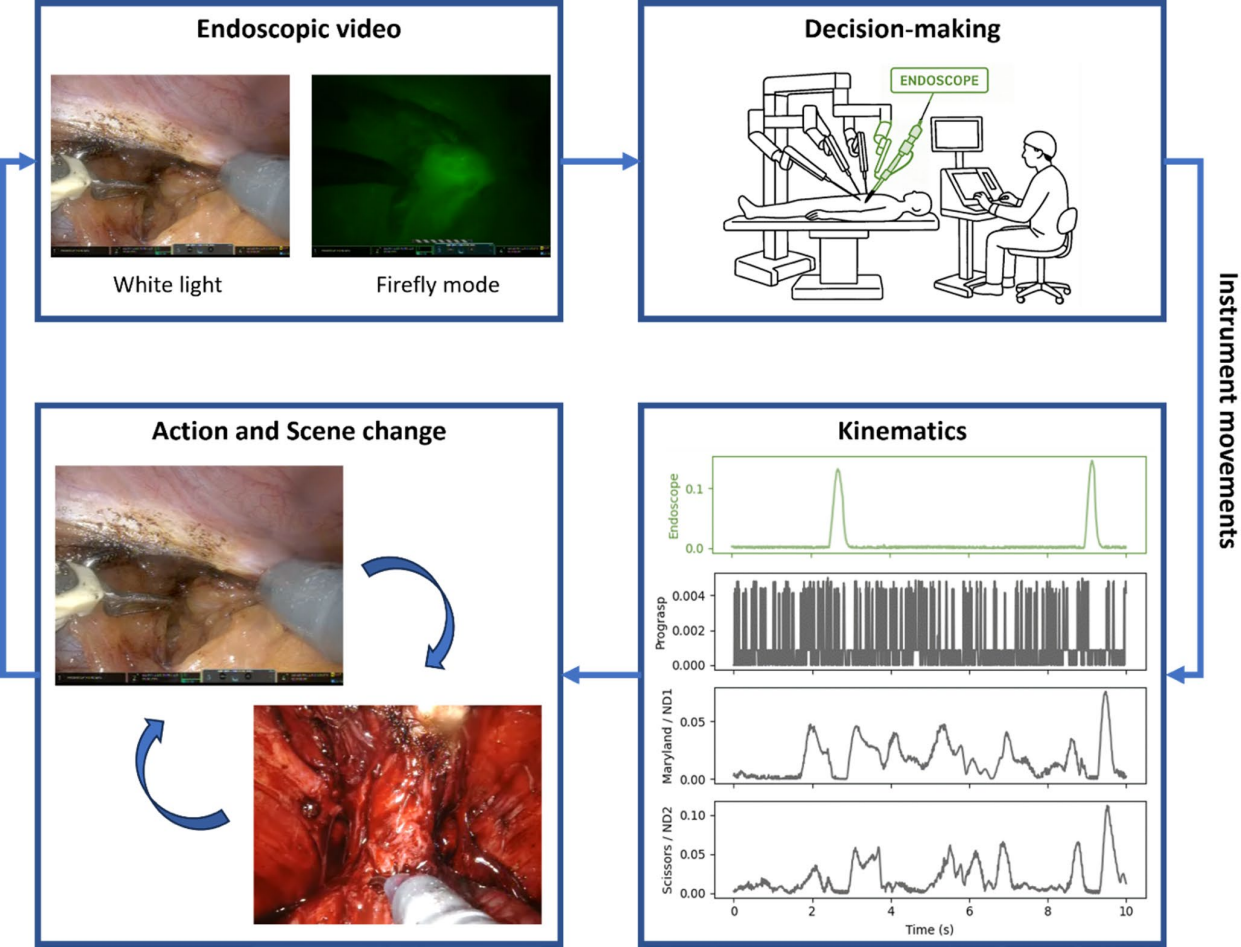
Conceptual relation of surgical vision to instrument kinematics. ND: Needle Driver

During minimally invasive surgery, the white light stereo-endoscope acts as an extension of the surgeon’s first-person (egocentric) vision, thereby driving hand-eye coordination. Unfortunately, the use of telerobotic instrument drivers, rather than the surgeon’s hands, limits visual-proprioceptive interpretation; tactile sensing is non-existent in older robotic platforms and remains limited to force-feedback in modern systems [4, 5]. As a result, vision is the dominant sensory channel through which a robotic surgeon interprets anatomy, identifies targets, and plans actions. In essence, what the surgeon sees or doesn’t see determines what she or he operates and how this is done [6]. This means that “vision” and “action” are intrinsically coupled with instrument movements and camera adjustments forming a continuous visuomotor loop (Fig. 1).

Recent advances in fluorescence-guided surgery have extended the perceptual capacity beyond white light vision e.g. via the da Vinci Firefly endoscope [7] or the HUGO RAS endoscope [8]. Fluorescence offers molecular and physiological cues that help advance anatomical interpretation in a way that exceeds human capacities. This enables new forms of image-guided decision-making to be clinically implemented [9]. Well-known examples in prostate cancer include the use of fluorescence during (sentinel) lymph node resections [10], anastomosis [11] and PSMA targeted surgery [12]. These ongoing efforts align with the broader goal of pursuing safer and more precise identification of tumors, tumor margins, and surrounding anatomical structures [13, 14]. Workflows wherein fluorescence intensities influence the surgeon’s decision-making capacities [15, 16] and where the mixing of white-light context with fluorescence target perception impacts the surgical utility of the fluorescence [10, 17, 18]. What is not yet known is if and how the implementation of fluorescence imaging influences endoscope and instrument kinematics.

Kinematic analysis of surgical instrument motion has proven to be a powerful method for quantifying technical skill and understanding surgical behavior [19–21]. Studies have used objective metrics such as path length, velocity, acceleration, and gesture patterns to assign steps [22], evaluate skill levels [19, 20, 23], and evaluate the impact of new technologies [24]. In most studies instrument actions are manually annotated [25] or tracked using endoscopic (stereo-) vision-based algorithms [26]. An exception is formed by studies employing robotic mechanical tracking, including the dVLogger [27] and AP4000 [22]. Despite the endoscope’s central importance in defining the surgical field of view and guiding instrument motion, the endoscope kinematics and their relation to other surgically relevant aspects remain unreported

In a pilot study using the AP4000 technology, we were able to gather detailed tracking information of the Firefly endoscope during a case series of prostate surgery. This allowed us to begin to unravel how endoscopic use relates to the resection. Specifically, we: (1) quantified the digitized endoscopic motion across the different surgical steps, (2) identified the frequency and effects of endoscopic cleaning, (3) studied the impact of fluorescence mode on the endoscope kinematics, and (4) related endoscopic vision (white-light and fluorescence) to kinematics of the dissecting instruments.

## Materials and methods

### Patient characteristics

In this study, 4 patients with localized prostate cancer underwent preoperative standard protocol diagnostics and staging and underwent a transperitoneal(+ robot-assisted radical prostatectomy, extended pelvic lymph node dissection) between 2023 and 2024. During surgery, an AP4000 mechanical tracking recorder (Intuitive Surgical Inc.) was used. The retrospective analysis of this data was approved by the local ethical committee (study number IRBd23 − 309), and the need for informed consent was waived.

## Surgical procedure

**Fig.2:**
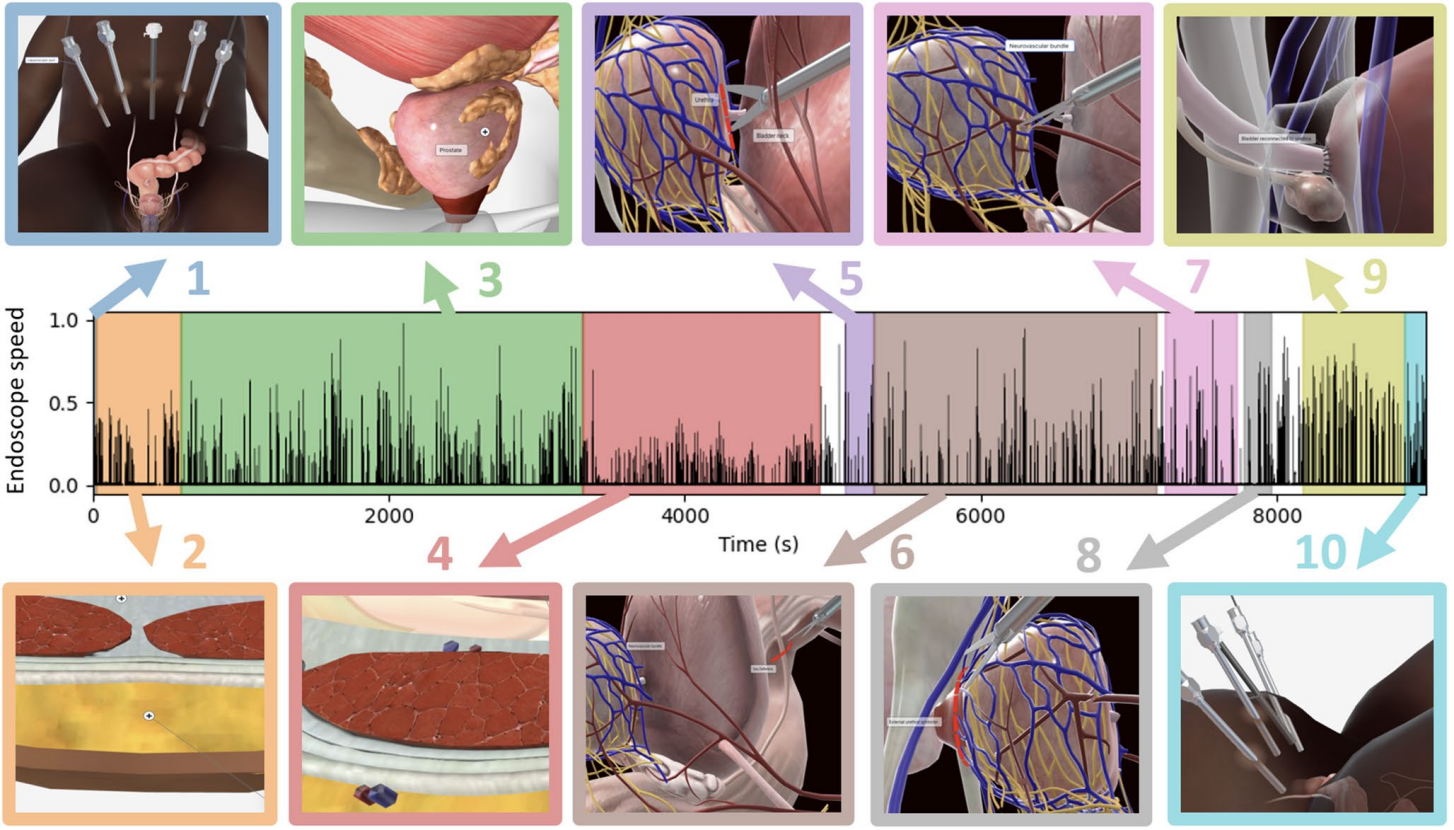
Variation in endoscope speed along color-coded RARP steps 1–10. Schematic endoscopic images illustrate each step on the sample procedure timeline, along with normalized speed of endoscope. White gaps on timeline indicate periods unrelated to defined surgical steps

Procedures were performed through a 6-port transperitoneal approach using the 4-arm da Vinci Xi system (Intuitive Surgical Inc.) by the same expert surgeon (HvdP). For all four procedures, the zero-degrees endoscope video output was manually annotated (HvdP, VO) to segment the timeline into ten distinct procedural steps (Fig. 2): (1) Preparation (blue), (2) Adhesiolysis (orange), (3) Lymph node dissection (green), (4) Retzius space dissection (red), (5) Anterior bladder neck transection (purple), (6) Posterior bladder neck transection; seminal vesicle (SV) and posterior dissection (brown), (7) Lateral and apical dissection (pink), (8) Urethral transection (gray), (9) Vesicourethral anastomosis (light green), (10) Final inspection and specimen extraction (cyan) [22, 25]. Firefly fluorescence imaging of indocyanine green (ICG) was applied during lymph node dissections (step 3) in 2 out of 4 procedures.

## Data preparation

Robotic arm movements in the x, y, and z (3D) dimensions were recorded using the research-grade AP4000 recording device that was attached to the surgical console to log the mechanical positions of instruments. Because we used mechanical tracking, no manual annotation was required.

Recordings were performed throughout the entire procedure at a sampling rate of 120 Hz. The video output from one “eye” (2D) of the endoscope was simultaneously captured at 30 Hz. To ensure temporal alignment, the digitized trajectories of all four robotic arms were synchronized with the endoscopic video stream. Each robotic arm was subsequently assigned to its corresponding instrument. Like our previous work [22], movement trajectories were used to extract 6 kinematic metrics, namely: (1) speed, (2) displacement, (3) distance to origin, (4) acceleration norm, (5) curvature, and (6) smoothness. For the velocity-related metrics, finite-difference formulas were applied to approximate derivatives with higher precision: $\mathrm{speed}=\parallel{v}_{k}\parallel$, displacement =$\:\:\parallel{p}_{k}-{p}_{k-1}\parallel$, velocity $\:{v}_{k}=\:\frac{{p}_{k+1}-{p}_{k-1}}{2\varDelta\:{t}_{k}}$, acceleration $\:{a}_{k}=\:\frac{{p}_{k+1}-2{p}_{k}+{p}_{k-1}}{{(\varDelta\:{t}_{k})}^{2}}$, acceleration norm =$\:\:\parallel{a}_{k}\parallel,\:$ curvature =$\:\:\frac{\parallel{v}_{k}\times\:{a}_{k}\parallel}{{\parallel{v}_{k}\parallel}^{3}}$, smoothness =$\:\:\frac{\parallel{p}_{k+2}-2{p}_{k+1}+2{p}_{k-1}-{p}_{k-2}\parallel}{2{(\varDelta\:{t}_{k})}^{3}}$, where p_k_ = (x_k,_ y_k,_ z_k_) and $\:\varDelta\:{t}_{k}$ is a local time step. Acceleration norm is further referred to as “acceleration” in the manuscript.

Each data entry included a timestamp representing the elapsed time since the start of the procedure, along with the corresponding 3D spatial coordinates of the robotic arm, the firefly mode state (ON/OFF), and the procedural steps as defined previously [22]. All data processing was conducted in Python 3.11.11 using pandas 2.2.2 and numpy 2.0.2.

## Statistics

Descriptive statistics are presented as means or median values with interquartile range. To quantify difference between surgical steps, we computed the straight-line (Euclidean) distance ($\:{d}_{ij}=\sqrt{\sum\:_{k}{({v}_{i,k}-{v}_{j,k})}^{2}}$) between the mean feature vectors of each step. Each vector included standardized 3D coordinates (x, y, z) and metrics for endoscope. Spearman’s rank correlation was used to assess relationships between kinematic metrics. Visualization was performed with matplotlib 3.10.0 and seaborn 0.13.2 libraries. Mann-Whitney U test was used for group comparison. Statistical tests were performed using scipy 1.16.1 library in Python 3.11.11. A p-value < 0.05 was considered significant.

## Results

### Endoscope motion across surgical steps

**Fig. 3:**
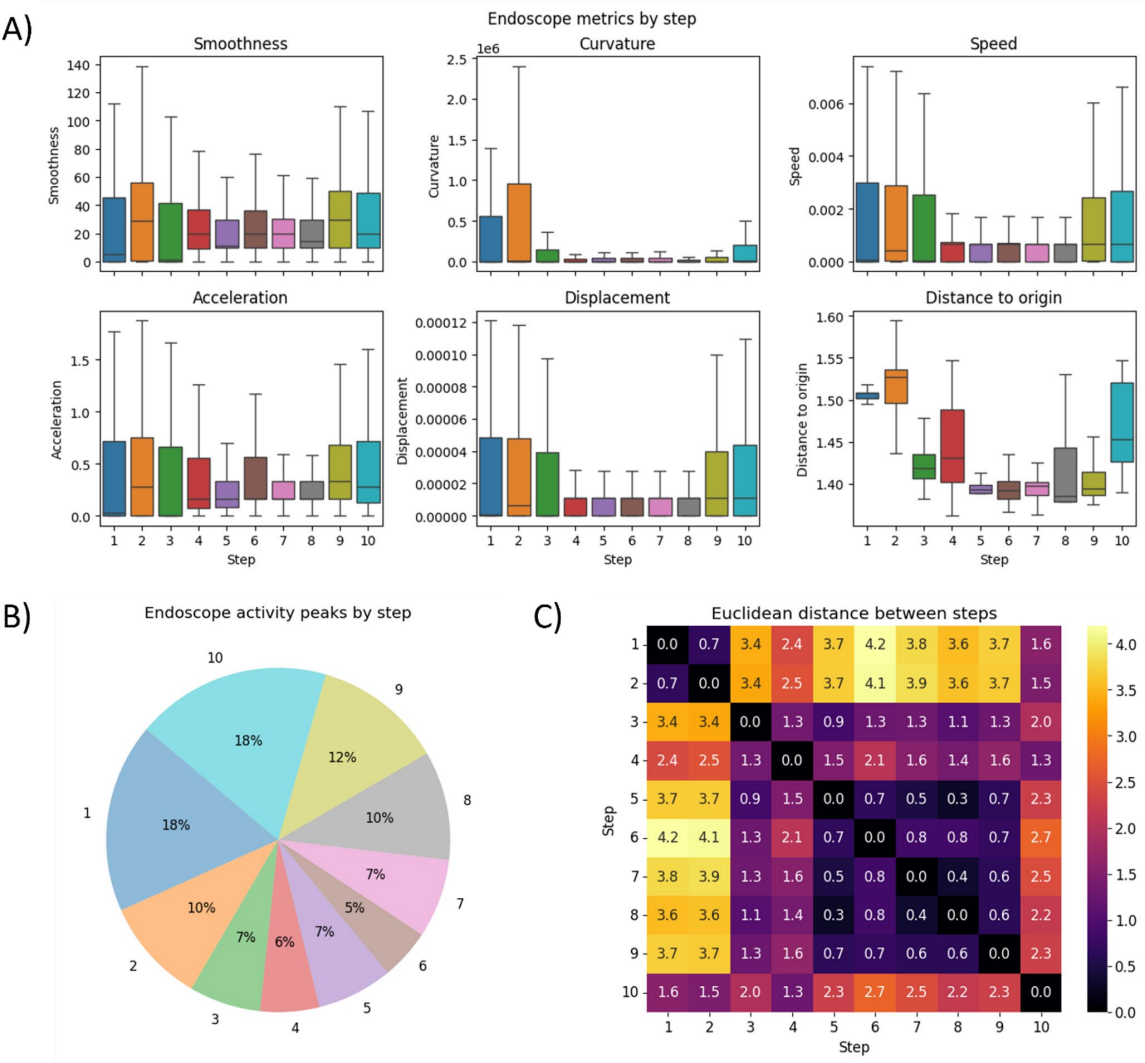
Endoscope movements through different steps of a sample procedure. (**A**) Boxplots of various kinematic metrics per step; (**B**) Pie chart of endoscope activity peaks (defined as peaks in displacement) per step corrected for step duration; (**C**) Difference between surgical steps calculated as Euclidean distance based on normalized endoscope kinematics and positional data alone (higher distance, marked by lighter color, corresponds to more different kinematic signatures between steps)

Endoscope kinematics significantly differed between the surgical steps. Mean endoscope curvature (8.4*10^8^-2.6*10^9^ vs. 3.3*10^8^-5.1*10^9^; *r* = 0.12), speed (2.6*10^− 3^-4.8*10^− 3^ vs. 3.4*10^− 3^-7.0*10^− 3^; *r*=-0.03), acceleration (2.9*10^− 1^-3.6*10^− 1^ vs. 3.2*10^− 1^-4.9*10^− 1^; *r*=-0.07) and displacement (2.8*10^− 5^-4.5*10^− 5^ vs. 3.5*10^− 5^-6.3*10^− 5^; *r*=-0.03) were generally lower (during the middle steps (steps 4–8 vs. steps 1–3, 5–10) of the procedure (Fig. 3A). A trend that reflects the execution of more precise surgical actions (i.e., Retzius, bladder neck, seminal vesicle, lateral/apical, and urethral dissection). Toward the end of the procedure (steps 5–9; e.g. bladder neck transection, seminal vesicle and posterior dissection, lateral/apical dissection, urethral transection, and vesicourethral anastomosis), distance to origin decreased compared to the early steps (1–4) (Fig. 3A). Indicating more deliberate and optimized endoscope movements. Overall endoscope activity, defined as peaks in displacement, was highest during steps 1, 2, 8, 9, and 10 (Preparation, Adhesiolysis, Urethral transection, Vesicourethral anastomosis and Final inspection and extraction, respectively; Fig. 3B). Steps that require exploratory maneuvers and navigation of complex anatomies.

The Euclidean distance shown in Fig. 3C represents the difference between step-averaged kinematic vectors, computed across all standardized features (speed, normalized acceleration, displacement, smoothness, curvature, positional coordinates), and therefore quantifies how distinct each step is from the others. In all four procedures the distance of the Preparation and Adhesiolysis (steps 1 and 2), as well as the Final inspection (step 10), were markedly different from the rest of the procedure. In all cases the distance observed in steps 5–9 was highly similar, consistent with the trends observed in the mean kinematic measures (Fig. 3A&B).

## Endoscope cleaning events

**Fig. 4:**
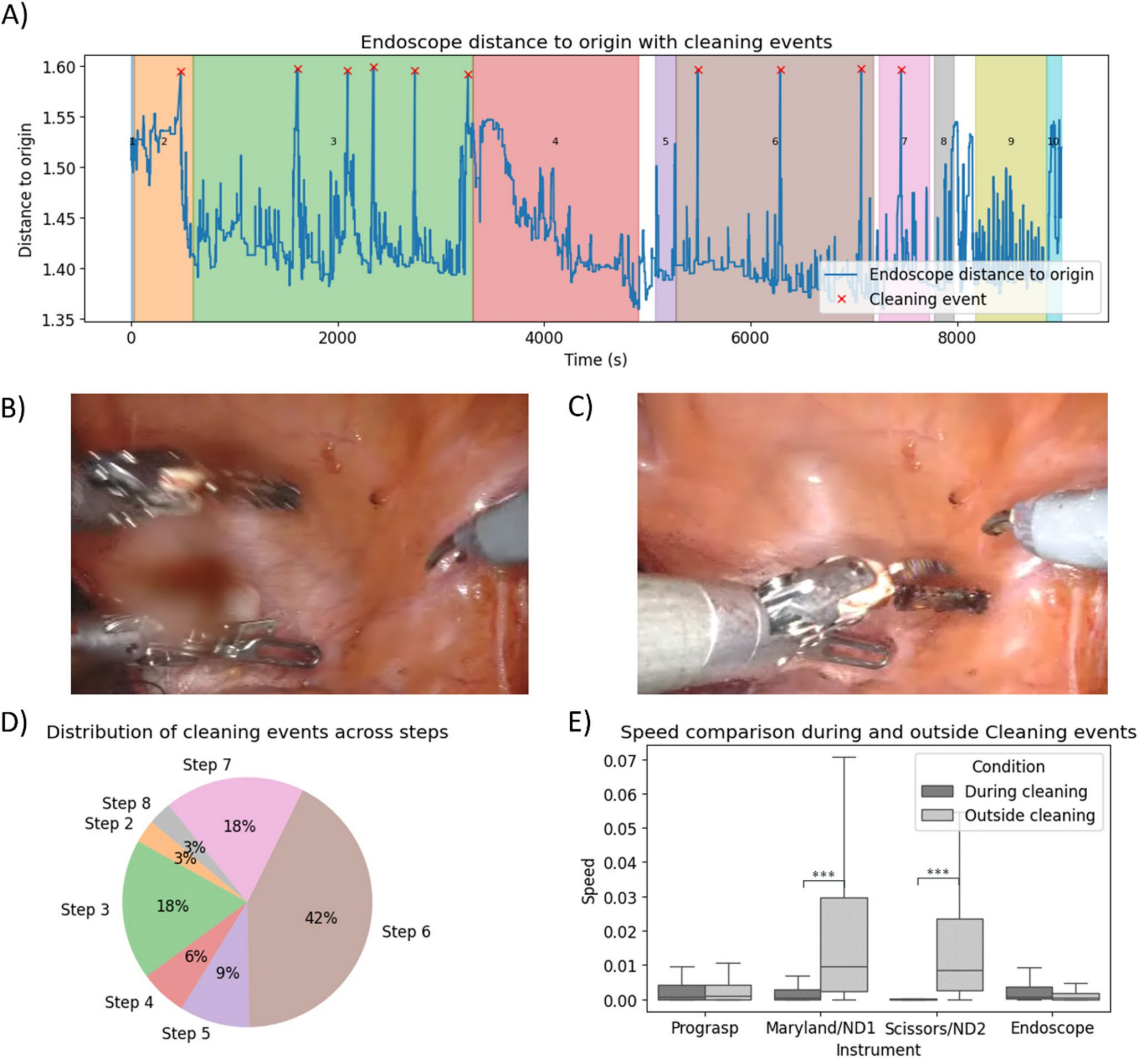
Cleaning of endoscope during surgical procedure: (**A**) Endoscope displacement to origin during the sample procedure with cleaning events marked with a x; (**B**) Example of a frame with dirty lens; (**C**) Example of the frame after lens cleaning; (**D**) Distribution of cleaning events across steps for all 4 surgeries combined; (**E**) Instrument speed comparison during and outside the cleaning events for the sample procedure. ND: Needle Driver

Removal of the endoscope for cleaning showed as sharp peaks in camera displacement and speed (Fig. 4A; Mean was 10 times per procedure). Cleaning occurred most frequently during steps 3, 6, and 7 (Lymph node dissection, Posterior bladder neck transection; SV and posterior dissection and Lateral and apical dissection; Fig. 4D). In these steps visibility was challenged due to lens fogging (Fig. 4B), or lens contamination with fluids (e.g. blood or urine). Each cleaning temporarily interrupted the surgical vision for 20.4 s. As the robot is designed to freeze instrument movements while the endoscope is repositioned, instrument motion was effectively paused during these out-of-body events. Accordingly, mean instrument speeds were significantly lower during endoscope cleaning compared with periods outside cleaning events (Fig. 4E). Specifically, reduced speeds were observed for the Maryland/Needle driver (ND): 10.0^− 3^ vs. 2.0*10^− 2^ (p-value < 0.05, *r* = 0.72), and the Scissors/ND2: 0 vs. 2.0*10^− 2^ (p-value < 0.05, *r* = 0.84). The small but non-zero instrument velocities observed in some cases (Fig. 4E) are likely attributable to brief mechanical jitter introduced during attachment or detachment of the endoscope from the robotic arm (Supporting Fig. 1).

## Impact of Firefly vision on endoscope kinematics

**Fig. 5:**
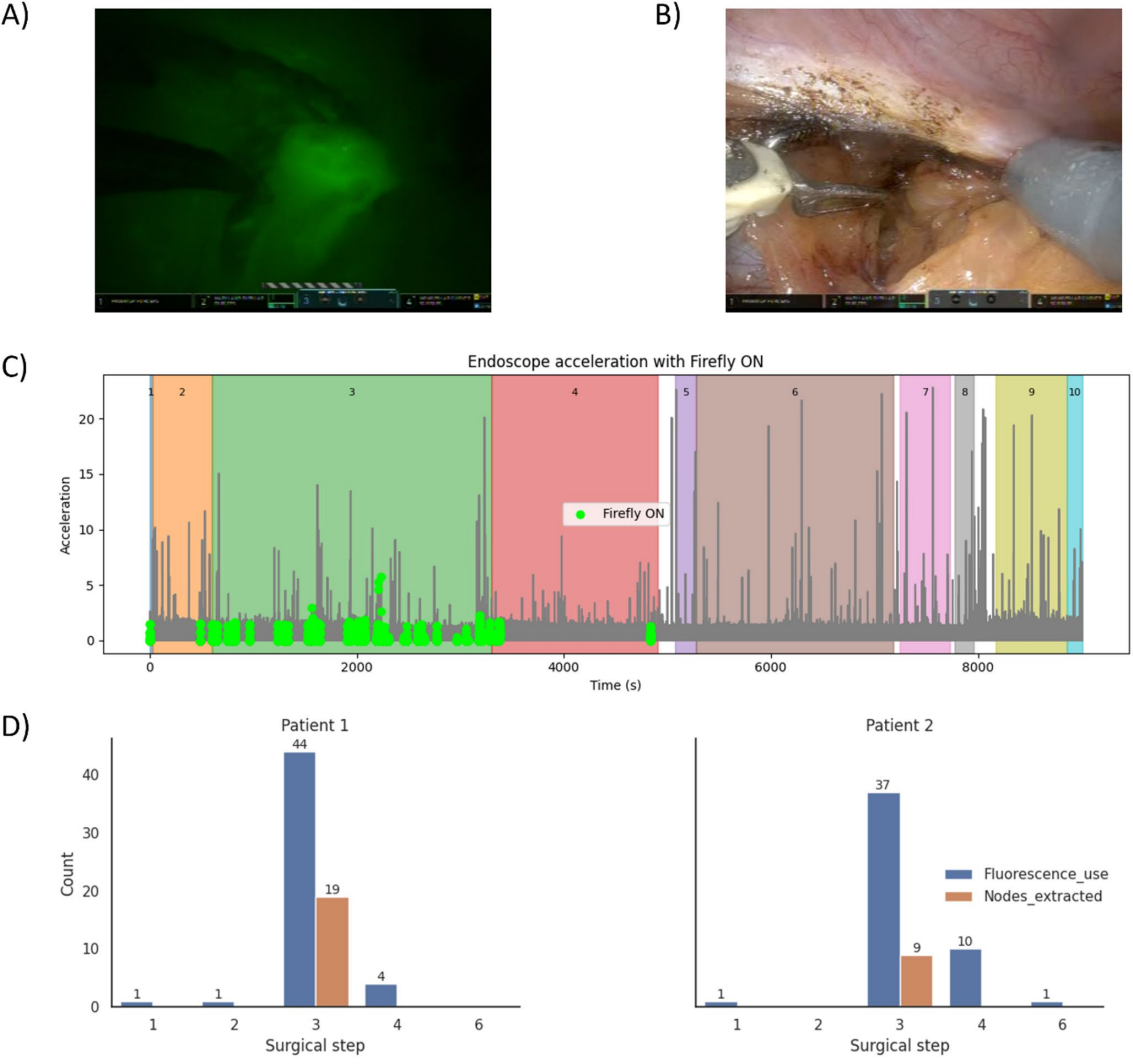
Impact of Firefly mode (one representative procedure shown): (**A**) Firefly mode ON showing an ICG-avid lymph node; (**B**) White-light image of the same lymph node, 1s apart; (**C**) Endoscope acceleration norm through the whole sample procedure with the use of Firefly marked in green; (**D**) The frequency of fluorescence use and the numbers of extracted lymph nodes per surgical step

In two patients, fluorescence imaging of ICG was used to guide lymph node removal (Fig. 5A-D; step 3). Beyond step 3, fluorescence imaging was also implemented during step 4 (Retzius space dissection) and steps 1, 2 and 6 (Preparation, Adhesiolysis and Posterior bladder neck transection; SV and posterior dissection; as seen in Fig. 5C-D). The operating surgeon (HvdP) indicated that these Firefly activations reflect intentional checking for draining lymph nodes. For example, nodes in the ventral bladder wall only become visible after opening of the space of Retzius.

Endoscope acceleration decreased 10-fold when the Firefly mode was activated (2.0*10^− 2^ vs. 1.7*10^− 1^, p-value < 0.05, *r*=-0.04; for Firefly mode and white-light, respectively; Fig. 5C), indicating slower, more gradual camera movements. Such motion patterns are consistent with the fine visual adjustments needed to preserve continuous visualization of fluorescent targets. During fluorescence mode, the endoscope tended to be pulled back or angled to maintain a wide, evenly illuminated field. This was reflected in endoscope’s distance to origin (1.42 vs. 1.4, p-value < 0.05, *r*=-0.3, for Firefly mode and white-light, respectively). In addition, the median displacement decreased (5.5*10^− 6^ vs. 8.5*10^− 6^, p-value < 0.05, *r*=-0.08), indicating that endoscope repositioning for individual targets decreased.

### Vision / action relationship

**Fig. 6:**
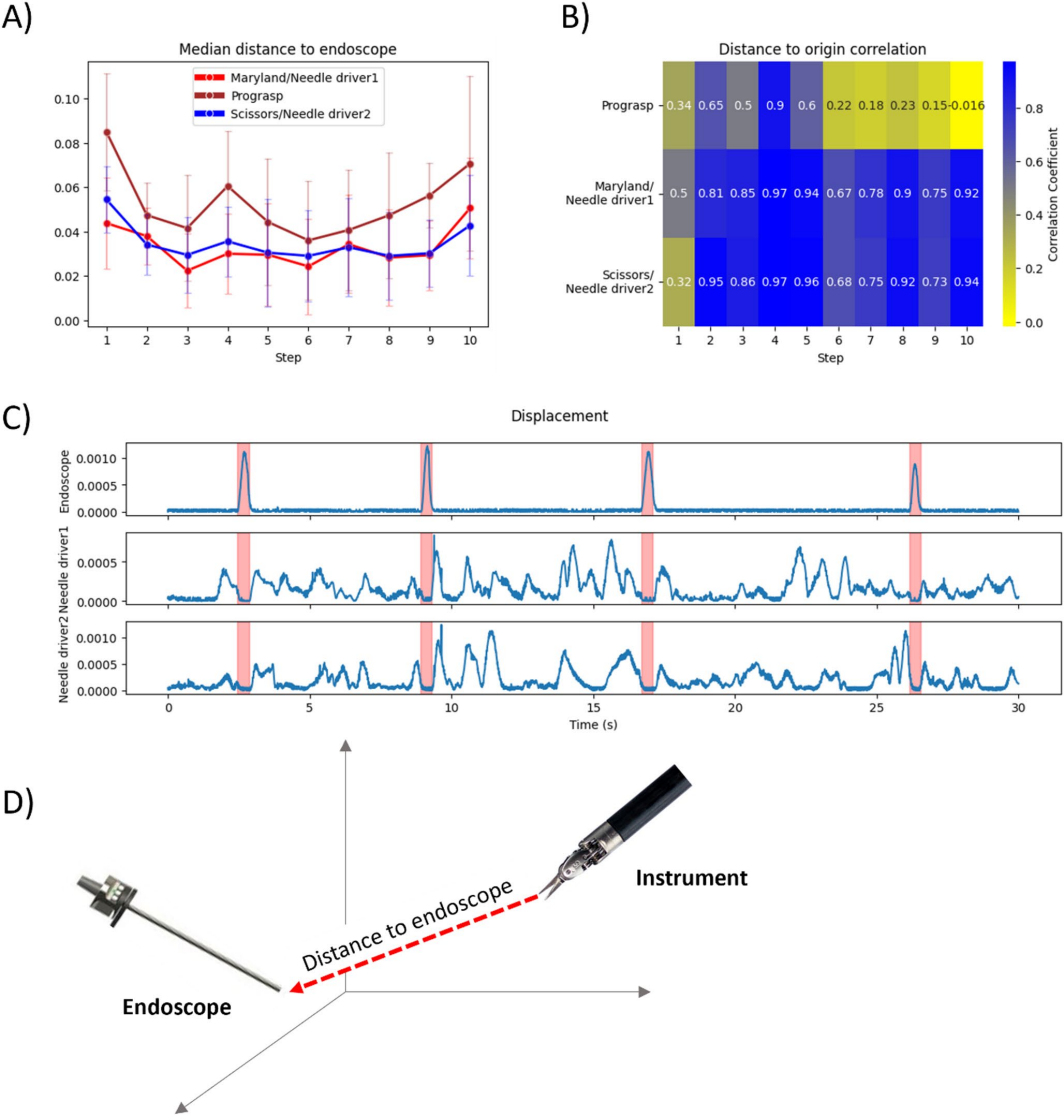
Correlation of endoscope with respect to the dissecting instruments (**A**) Median and standard deviation of the distances between endoscope and other instruments across surgical steps, averaged for all four procedures; (**B**) Correlation heatmap between distance to origin of endoscope and other instruments across steps for the sample procedure; (**C**) Distance to origin during first 30 s of anastomosis for Endoscope and Needle drivers in the sample procedure; endoscope repositioning events marked in red; (**D**) Schematic figure of distance to endoscope

Camera control reflected the coordinated activity of the dissecting instruments within the same anatomical space. Patterns observed in Fig. 6A-B align to the progressive refinement of endoscope movements. As the endoscope becomes more precise and stable (reflected in lower curvature, speed, acceleration and displacement, as seen in Fig. 3A), the main dissecting instruments (Maryland, Scissors, Needle drivers) maintain tighter and more coordinated trajectories within the operative field. For example, throughout steps 2–9. Strong positional correlations between the endoscope and dissection instruments (Fig. 6B, D) could be observed throughout steps 2–10. In contrast, the Prograsp instrument, often used to hold tissue flaps, showed more distant movements (steps 1, 4, 8–10; Fig. 6A) and more independent movements over time (steps 6–10; Fig. 6B, D).

Endoscope adjustments prevent surgeons from manipulating the tissue. In line with this, instrument motion paused during camera movements and vice versa (Fig. 6C highlighted in red).

In response to switching to fluorescence mode, the Prograsp instrument was moving faster (9.1*10^− 4^ vs. 4.8*10^− 4^ fluorescence vs. white light, respectively, p-value < 0.05, *r*=-0.08) with higher acceleration (7.0*10^− 1^ vs. 3.0*10^− 1^, p-value < 0.05, *r*=-0.11) and larger displacement (1.05*10^− 5^ vs. 7.5*10^− 6^, p-value < 0.05, *r*=-0.08), as well as higher smoothness (6.3*10^1^ vs. 2.9*10^1^, p-value < 0.05, *r*=-0.13). Maryland had higher displacement (7.7*10^− 5^ vs. 7.1*10^− 5^, p-value < 0.05, *r* = 0.01) and acceleration (4.0*10^− 1^ vs. 3.0*10^− 1^, p-value < 0.05, *r*=-0.04), lower curvature (1.6*10^3^ vs. 1.8*10^3^, p-value < 0.05, *r* = 0.01) and higher smoothness (2.7*10^1^ vs. 1.6*10^1^, p-value < 0.05, *r*=-0.03). For the Scissors instrument, no significantly different motion kinematics were observed between fluorescence and white light modes. Combined, these findings may reflect differences in instrument handling associated with fluorescence-guided exploration, especially noticeable for the Prograsp and Maryland graspers.

## Discussion

This pilot study highlights how the endoscope dynamics vary throughout a typical robot-assisted prostate cancer procedure. Just like what was previously reported for the dissecting instruments [22], the way the endoscope is manipulated seems to follow identifiable patterns that vary depending on the surgical step and task. In particular, episodes of lens cleaning stand out when compared with other endoscopic movements (Fig. 4). These events temporarily halt instrument activity, clearly marking interruptions in surgical workflow. Furthermore, early and late procedural steps tend to involve broader exploratory endoscopic movements. Conversely, procedural steps requiring fine dissection or precision show slower, more deliberate endoscope adjustments. This suggests that camera control reflects the perceptual demands of different steps in the procedure.

Eye-tracking is widely considered to be a generic performance indicator in aviation [28], Formula 1 [29] as well as surgery [30]. During laparoscopic surgery, for example, randomized trials have indicated that psychomotor task performance, including unexpected events, depends on surgeons’ gaze patterns [31, 32]. We argue that, because robotic surgeons can freely reposition the endoscope - a surrogate for their own eyes - endoscope tracking provides a practical alternative to eye‑tracking. In line with this reasoning, we observed that endoscope kinematics are closely related to psychomotor task demands. When precise and synchronized manipulation is needed, the endoscope seems to have a higher positional correlation with the primary dissecting instruments (Maryland, Scissors, Needle drivers). In contrast, the supporting Prograsp instrument shows a weaker positional correlation, which could reflect the instrument’s more independent or ancillary role. Taken together, these observations indicate a functional coupling between endoscope kinematics and dissecting instrument movements throughout the procedure (Fig. 6).

Switching to fluorescence mode provides a clear indication of how changes in surgical goals and visual perception alter the use of vision. It is important to note that fluorescence is used to support visual exploration of lymph node targets. At the same time, the white light context is lost, which is known to negatively impact the surgeon’s perception [10, 17, 18]. Irrespective of the cause, our findings indicate that the surgeon adapts to the fluorescence workflow by widening the field of view and slowing down endoscope movements (e.g. increased stability and reduced repositioning), which is also reflected in instrument kinematics (e.g. timing and coordination of instrument activity). While one would expect the use of fluorescence to be confined to step 3 (lymph node removal), our analysis revealed that fluorescence was also intentionally implemented in earlier and later steps. While fluorescence use in earlier steps was intended to support guidance during step 3, its use in later steps served to verify whether deeper‑lying fluorescent lymph nodes - whose signals may be attenuated by overlying tissue - had been missed during step 3.

From a future perspective, endoscopic tracking may serve several purposes. The feasibility data presented suggests it could help provide insight into the eye-hand coordination of expert robotic surgeons. This knowledge could subsequently be used for surgeon self-evaluation or performance assessment. Improved understanding of which endoscopic features are associated with expert performance may help refine proficiency assessments for novice surgeons in training. In this context, the relationship between instrument ergonomics and endoscopic vision suggests that targeted training in camera control may help trainees optimize instrument handling. This assumption, however, requires further validation. Going one step further, we argue that improved insights into the use of endoscopic vision will also be essential for future surgical robotic automation attempts. This concept is gaining traction [33, 34], but also requires comprehensive digitization of the multidimensional aspects of surgeon-patient interactions. While this work by no means supports robotic automation, the digitization of expert use of endoscopic vision does provide a first step toward understanding which type of vision, under what angles etc. is needed for a human to accurately perceive the surgical field.

Reliance on the prototype AP4000 logger limited the analyses to 4 surgeries, only half of which employed fluorescence. As such, this limitation constrains the impact of our pilot findings. Before clear performance-related conclusions can be drawn, a broader patient body needs to be monitored. Similarly, behavioral parameters derived from a single surgeon are difficult to generalize, highlighting the need to diversify the surgeon cohort, ideally supported by expert consensus-based proficiency assessments [35]. The new da Vinci 5 platform includes embedded kinematic analysis that is accessible through a dedicated application [36]. How this information is collected is unknown, but this could well be based on a mechanical logger. If so, extension of both patient cohorts and user populations may primarily be limited by the number of installed da Vinci 5 systems.

In the absence of mechanical tracking, evaluation of endoscopic kinematics becomes considerably more challenging. This will require integration of alternative tracking technologies to fully capture endoscope-instrument dynamics across different surgical set-ups. Examples include external endoscope tracking [37] and vision-based endoscope tracking [38], each facing their own technical challenges.

## Conclusions

The feasibility data obtained suggest that endoscope movements vary with surgical perceptual demands and are coordinated with the movements of dissecting instruments, thereby providing a new avenue for monitoring and evaluating surgical performance.

## Supplementary Information

Below is the link to the electronic supplementary material.

Supplementary Material 1

## Data Availability

Data is available upon reasonable request.
