# Supplementary material for: Eye-hand coordination during robotic prostate cancer surgery – how surgical vision reflects on instrument kinematics and vice versa
Source: J Robot Surg. 2026 May 28;20(1):548. doi: 10.1007/s11701-026-03499-y (PMC13216139; doi:10.1007/s11701-026-03499-y)
Supplement: Supplementary file 1 — Supplementary Material 1 [file 11701_2026_3499_MOESM1_ESM.docx]

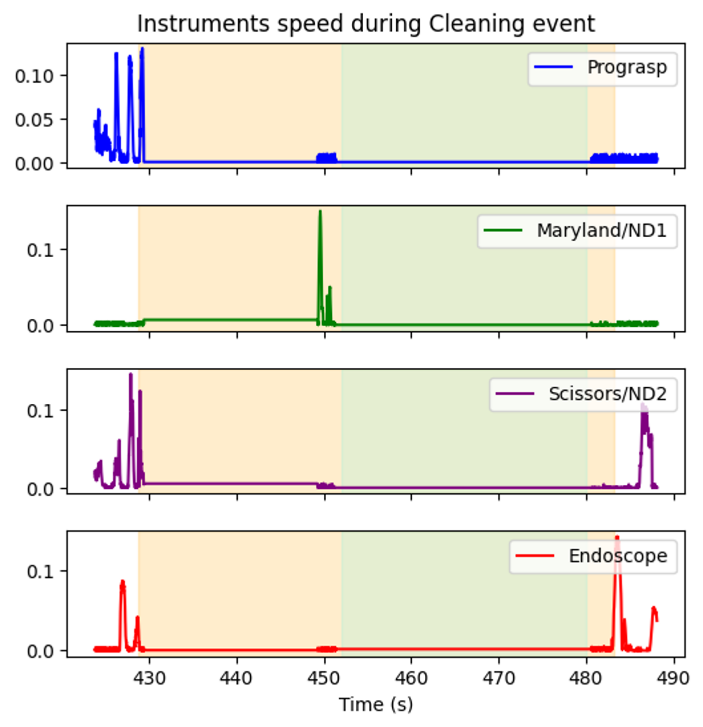
**Supporting Figure 1.** Zoom in investigation of instruments’ speed during example cleaning event with cleaning margins, indicating endoscope movements through the trocar (light yellow) and outside of body margins (light green).
